# First molecular detection of *Neospora caninum* from naturally infected slaughtered camels in Tunisia

**DOI:** 10.1002/vms3.901

**Published:** 2022-08-16

**Authors:** Yosra Amdouni, Imen abedennebi, Safa Amairia, Amara Abdelkader, Walid Chandoul, Mohamed Gharbi

**Affiliations:** ^1^ Laboratoire de Parasitologie University Manouba Institution de la Recherche et de l'Enseignement Supérieur Agricoles École Nationale de Médecine Vétérinaire de Sidi Thabet Sidi Thabet Tunisia; ^2^ Laboratoire d'anatomie Pathologique École Nationale de Médecine Vétérinaire de Sidi Thabet Sidi Thabet Tunisia; ^3^ Ministry of Agriculture Water Resources and Maritime Fisheries, Arrondissement de Production Animale de Médenine Médenine Tunisia

**Keywords:** camelids, molecular detection, *Neospora caninum*, PCR, South Tunisia

## Abstract

**Background:**

*Neospora* *caninum* has been documented to infect most domestic wildlife but is known to primarily infect dogs and cattle and is considered an important cause of abortion in camels.

**Objective:**

The aim of this study was to estimate the molecular detection of *Neospora caninum* in tissues of naturally infected camelids.

**Methods:**

Brain, tongue (bottom and tip) and masseter muscles from 35 slaughtered camelids from Tataouine and Médenine regions were collected (*n* = 140 samples). PCR was used to amplify and detect *N. caninum* DNA in tissues samples followed by sequencing of some PCR products. A phylogenetic tree was then constructed to compare the partial sequences of the ITS1 gene with GenBank sequences. Histopathology examination was used to detect *Neospora spp*. cysts, but no lesions were observed.

**Results:**

The overall molecular detection of *N. caninum* in camelids was 34.3% (12/35).

The highest molecular detection of *N. caninum* was recorded in animals of more than 3 years old (6/9) and in animals aged between 1 and 3 years old (4/12). Whilst, the lowest molecular detection (2/14) was observed in animals 1 year or younger (*p* = 0.035).

There were no significant differences in molecular detection of *N. caninum* according to both locality and gender (*p* > 0.05). Similarly, there was no difference of prevalence between different anatomical locations. Comparison of the partial sequences of the ITS1 gene revealed 100–95.5% similarity among our *N. caninum* amplicon (MW551566) and those deposited in GenBank.

**Conclusion:**

These results highlight the presence of a risk infection by *N. caninum* in camels. For preventing *N. caninum* infection further studies are needed to improve our knowledge about the epidemiology of neosporosis in North Africa.

## Graphical abstract

In Tunisia camels represents the first animal production of arid zones, with poor and/or halophyte vegetation. Despite their social and economic importance in remote Tunisian Saharan region, little is known about camel diseases and there are no data about the molecular detection of N. caninum in camels in Tunisia. The present study is the first to detect of N. caninum DNA in Tunisian dromedary camels slaughtered in the regional slaughterhouse of Médenine and Tataouine (Southern Tunisia).

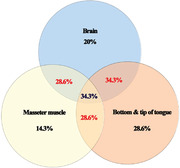

## 1 INTRODUCTION

Camels represent the first animal production of arid zones, with poor and/or halophyte vegetation. In Tunisia, the population of one‐humped camels (*Camelus dromedarius*) was estimated as 80,000 productive females (OEP, 2021). Despite their social and economic importance in remote Tunisian Saharan region, little is known about camel diseases.

In Tunisian camels, abortive diseases are a major health problem since the camel population is low with a high consanguinity. Among these abortive diseases, neosporosis, is caused by an intracellular protozoan parasite (*Neospora caninum*). It is regarded as one of the main abortive pathogens in cattle worldwide (Almería & López‐Gatius, 2013; Dubey, 2003; Dubey et al., 2007) inducing high economic losses in livestock industry (Reichel et al., 2013). Neosporosis could result in embryonic resorption, early embryonic death, abortion, foetal death with mummification, still birth, birth to congenitally infected but healthy animals, and neonatal deaths (Bártová et al., 2017; Hässig et al., 2003; McAllister et al., 1996; Moreno et al., 2012). In dogs, the definitive hosts, it can cause neuromuscular disease and death (Dubey et al., 2017). The main intermediate host of *Neospora caninum* is cattle but infection has been detected in many other domestic mammals, such as dogs, sheep, goats, horses (Dubey, 1999; Dubey & Schares, 2011; Donahoe et al., 2015), antelope (Peters et al., 2001), alpacas and llamas (Chávez‐Velásquez et al., 2004; Wolf et al., 2005) and in one‐humped camels (Hilali et al., 1998; Hosseininejad et al., 2009; Sadrebazzaz et al., 2006).

Anti‐*N. caninum* antibodies in camels were detected in several regions in the world with a seroprevalence that varies between 3.7 and 86% (Selim & Abdelhady, 2020). Serological studies were carried out in Egypt (Hilali et al., 1998; Selim & Abdelhady, 2020), Pakistan (Nazir et al., 2017), Iran (Sadrebazzaz et al., 2006), Centre of Iran (Hamidinejat et al., 2013), Spain (Mentaberre et al., 2013), Czech Republic (Bártová et al., 2017), Kingdom of Saudi Arabia (Mohammed et al., 2020) and Sudan (Ibrahim et al., 2014).

As far as we know, there are no data about the molecular detection of *N. caninum* in camels in Tunisia. The present study is the first to detect of *N. caninum* DNA in Tunisian dromedary camels slaughtered in the regional slaughterhouse of Médenine and Tataouine (Southern Tunisia).

## 2 MATERIALS AND METHODS

### 2.1 Study area and specimen collections

**FIGURE 1:**
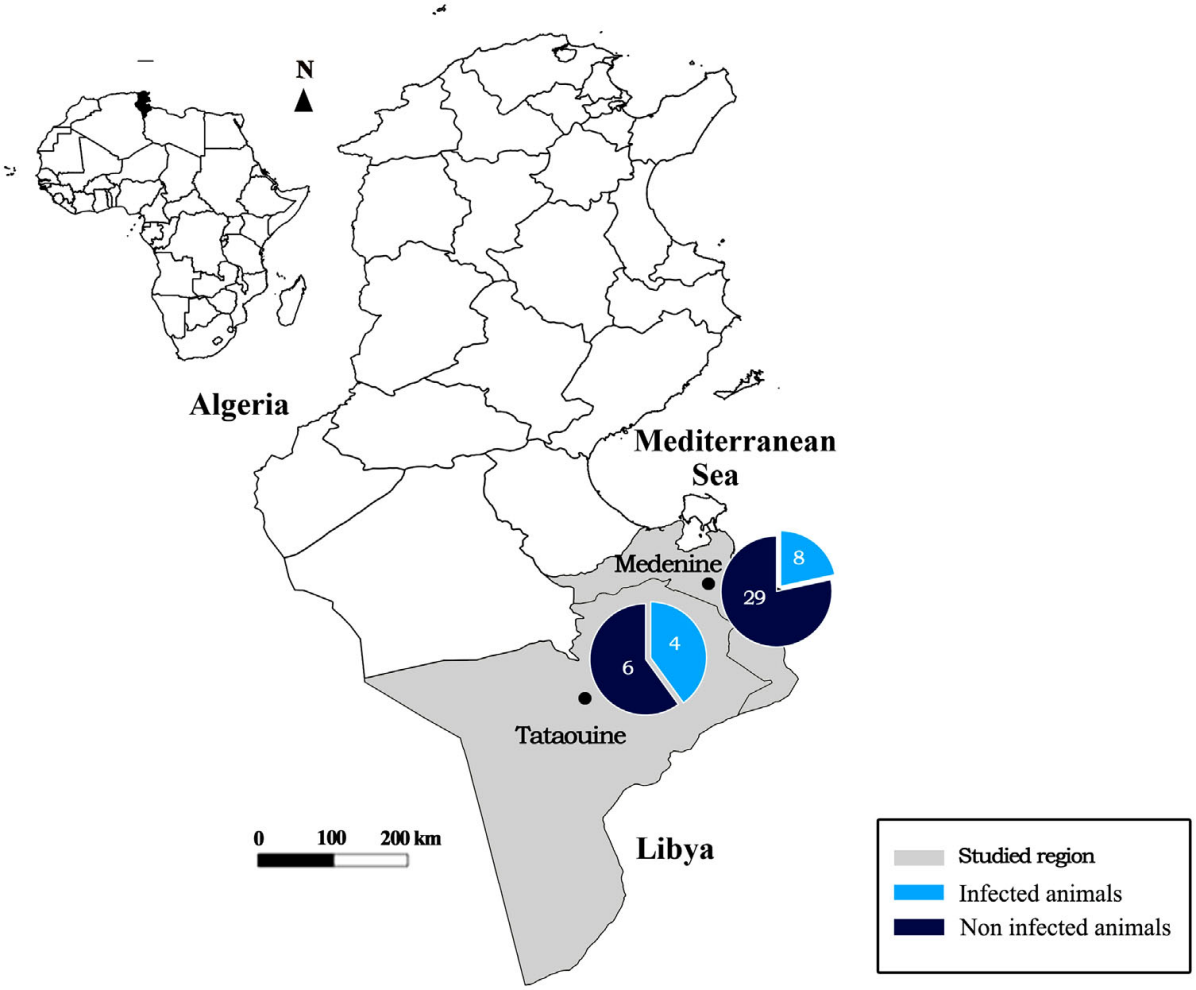
Molecular detection of *Neospora caninum* infection rates in tissues of naturally infected camelids sampled from Tataouine and Médenine slaughterhouse (South Tunisia)

Tissue samples were collected from 35 camels presented for slaughter at the regional slaughterhouses of two governorates from south Tunisia: districts of Médenine and Tataouine characterized by arid and Saharan climate, respectively (Figure 1; Table 1). In Médenine district, the mean temperature in winter and summer is 12 and 27.7**°**C, respectively. The mean annual rainfall is 156 mm with very large interannual variations. In Tataouine district, the mean temperature in winter and summer is 11.2 and 27.7**°**C, respectively. The mean annual rainfall is 134 mm with very large interannual variations. The camel population in the two governorates included in the present study is estimated to be 12,293 and 11,000, respectively. http://www.ods.nat.tn/fr/index.php?id=32

**TABLE 1:** Geographic and abiotic characteristics of the study regions (https://fr.climate‐data.org/)

District	Number of meat samples	Bioclimatic zone	Mean altitude (m)	Mean temperature (°C)		Annual temperature (°C)	Mean annual rainfall (mm)
				Winter	Summer	Min–Max	
Médenine	116	Arid	103	12.1	27.7	11.3‐29	156
Tataouine	24	Saharian	237	11.2	27.7	10.2‐29.5	134

One hundred and forty tissue samples were collected from the bottom and tip of the tongue, masseter muscle and brain of 35 slaughtered dromedaries belonging to different age groups and both genders.

Samples were collected in sterile and identified bags, transferred to the laboratory of parasitology, National School of Veterinary Medicine of Sidi Thabet, Tunisia and stored at −20°C until processed.

### 2.2 DNA extraction

After thawing the samples, for each tissue sample location, 50 mg of tissue was cut using a sterile disposable blade for DNA extraction. Each tissue sample was washed with sterile distilled water and centrifuged at 16,000 *g* for 6 min. DNA was extracted using Wizard Genomic DNA purification kit (Promega, Madison, Wisconsin, USA) according to the manufacturer's instructions then stored at −20°C until analysed.

To appreciate the quality of DNA in each extract prior to PCRs, universal PCR was performed for each sample using forward and reverse primers 1A and 564R (Table 2) targeting the hypervariable regions V1‐V3 coding for 18S rRNA (Wang et al., 2014). PCRs reactions were carried out with a mix consisting of 1× PCR buffer, 2 mM MgCl_2_, 10 μM of each primer (1A and 564R), 0.2 mM of each dNTP, 2 U Taq polymerase (Vivantis, Chino, California, USA), 1.5 μL of DNA template and distilled water in a total volume of 25 μL. Amplification was done in the following conditions: initial denaturation at 94°C for 5 min followed by 25 cycles (94; 59 and 72°C for 50 s each) and a final extension at 72°C for 10 min and a 4°C hold at the completion of the profile (Wang et al., 2014).

**TABLE 2:** Association between *Neospora caninum* molecular infection prevalence and different parameters based on PCR DNA amplification

Factor	Parameter	Positive/examined	OR [95% CI]	*p‐*Value
Locality	Médenine	8/29	0.02 [0.19; 1.61]	0.06
	Tataouine	4/6		
Age group (years)	≤1	2/14	0.34 [0.02; 3.1]	0.035*
	]1–3] <1 and ≥3	4/12		
	>3	6/9		
Gender	Male	9/27	0.8 [0.13; 6.62]	1
	Female	3/8		

Abbreviations: 95% CI, 95% confidence interval; NA, not applicable; OR, odds ratio.

*p ≤ 0.05.

### 2.3 PCR amplification of the *ITS1* gene of *N. caninum*

A nested PCR was performed with four oligonucleotides to amplify a 279 bp *N. caninum* DNA fragment belonging to *ITS1* gene and coding for the 18S‐5.8S rRNA according to the protocol of Buxton et al. (1998).

A primary PCR was performed with 0.15 μM of each primer (NN1 and NN2) (Table S1) in a total reaction volume of 25 μL consisting of 3 μL of DNA sample, 1x PCR buffer, 2.5 mM MgCl_2_, 200 μM dNTP each, 1 U of Taq Polymerase. The amplification was carried out in a thermocycler under the following cycling conditions: 95°C for 5 min, followed by 26 cycles (denaturation at 94°C, annealing at 48°C and extension at 72°C for 1 min each) and a final extension at 72°C for 5 min. We added 2 μL of the amplicons as template for the second PCR using the same mixture as primary PCR and 0.2 μM of each inner primer NP1 and NP2 (Table 1) and amplified for 26 cycles of 60 s at 94°C, 30 s at 48°C and 30 s at 72°C, with final extension cycle increased to 5 min. Positive and negative controls consisting of *N. caninum* DNA and nuclease free water were added for each PCR run, respectively. The amplicons were visualized by electrophoresis in 1.8% (w/v) agarose gel mixed with 0.05% ethidium bromide in TAE buffer.

A dromedary was considered *N. caninum*‐infected if at least one samples was PCR positive.

### 2.4 DNA sequencing and phylogenetic analysis

Five positive amplicons of *N. caninum* were randomly selected for sequencing and phylogenetic analysis. These amplicons were purified using the ExoSAP‐IT (Affymetrix Inc., USA) DNA clean‐up kit, according to the manufacturer's instructions. The purified PCR products were sequenced in both directions with the two PCR primers (NP1 and NP2), using the BigDye Terminator v3.1 Cycle sequencing chemistry (Applied Biosystems, ThermoFisher) in an ABI Prism 3500 DNA analyser. The chromatograms were evaluated with ChromasPro software (version 1.7.4) and compared with published sequences on the National Center for Biotechnology Information (NCBI) database using the Basic Local Alignment Search Tool (BLAST). MEGA 5 software was used as described by Tamura et al. (2011), to perform multiple sequence alignments. A phylogenetic tree was constructed using the Neighbour‐joining (NJ) algorithm (Saitou & Nei, 1987) as implemented in MEGA 7 following 1000 bootstrap replications (Figure S1).

### 2.5 Histopathological examination

Small tissue samples (0.5 × 0.5 cm) from the tongue, masseter muscle and brain were collected and fixed in 10% neutral buffered formalin for 48 h. They were dehydrated in graded alcohol series, cleared in toluene then processed by the standard paraffin embedding technique. The slices were cut at 4 μm thick, and mounted on microscope slides. They were stained with hematoxylin and eosin (HE) and finally examined under an optical microscope at 400× then 1000× magnifications for the detection of *Neospora* spp. cysts.

### 2.6 Statistical analysis

Descriptive statistics, including molecular prevalence of *N. caninum* infection in each location, age group and gender were estimated. One dromedary was considered *N. caninum*‐infected if at least one of the samples was PCR positive. Differences in molecular prevalence for all parameters were analysed by chi square Mantel–Haenszel test with Epi Info 6 software at 5% threshold (Schwartz, 1993). Odds ratios were estimated in each animal group (Ancelle, 2006).

## 3 RESULTS

### 3.1 Molecular detection of *N. caninum*

All tested samples were positive for 18S rRNA universal PCR. Out of 35 tested animals, 12 were *N. caninum* PCR‐positive corresponding to an overall molecular detection of 34.3%.

The highest molecular detection of *N. caninum* was recorded in animals of more than 3 years old (6/9) and in animals aged between 1 and 3 years old (4/12), whereas the lowest molecular detection (2/14) was observed in animals 1 year or younger (*p* = 0.035).

**FIGURE 2:**
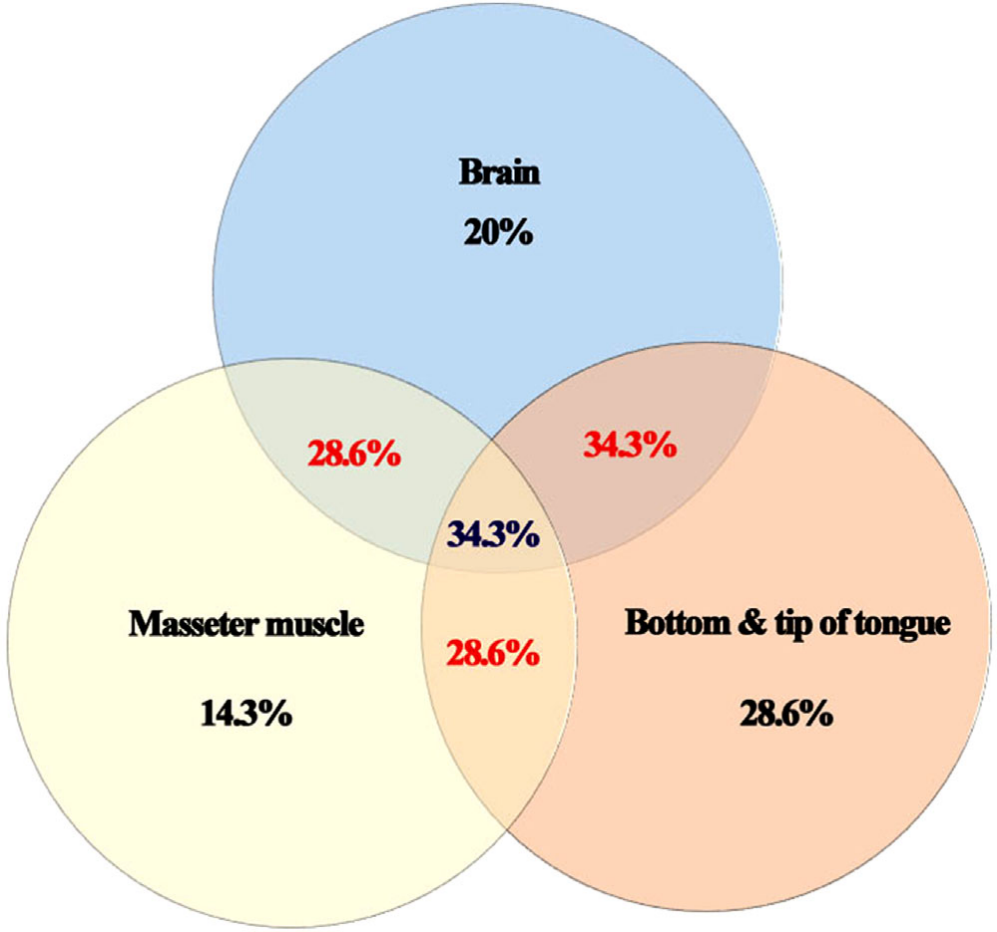
Venn diagram of interaction between *Neospora caninum* molecular infection prevalence in different categories samples

There were no significant differences of *N. caninum* molecular detection according to both locality and gender (*p* > 0.05). Similarly, there was no difference of prevalence between different anatomical locations (Figure 2) (Tables 2 and 3).

**TABLE 3:** Association between *Neospora caninum* molecular infection prevalence in different tissue's samples and parameters based on PCR DNA amplification

	Positive/examined	OR [95% CI]	*p‐*Value	Positive/examined	OR [95% CI]	*p‐*Value	Positive/examined	OR [95% CI]	*p‐*Value	Positive/examined	OR [95% CI]	*p‐*Value
Parameter	Tip of the tongue			Bottom of the tongue			Masseter muscle			Brain		
Locality												
Médenine	4/29	0.06 [0.8; 23]	0.85	5/29	0.04 [0.4; 4.4]	0.37	5/29	NA	0.27	5/29	0.04 [0.4; 4.4]	0.37
Tataouine	1/6			2/6			0/6			2/6		
Age group (years)												
≤1	0/14	NA	0.08	1/1	0.01 [0.15; 2]	0.24	1/14	1.01 [0.4; 6.8]	0.57	1/14	1.15 [0.4; 2]	0.24
]1‐3]	2/12			4/12			2/12			4/12		
>3	3/9			2/9			2/9			2/9		
Gender												
Male	4/27	0.09 [1.2; 33.6]	0.87	6/27	0.17 [2; 52]	0.55	5/27	NA	0.19	6/27	0.01 [0.15; 2]	0.55
Female	1/8			1/8			0/8			1/8		

Abbreviations: 95% CI, 95% confidence interval; NA, not applicable; OR, odds ratio.

*p ≤ 0.05.

### 3.2 Phylogenetic analysis of *N. caninum*

All blasted amplicons were 100% identical; therefore, only one sequence was submitted to NCBI GenBank under the accession number MW551566. A phylogenetic tree of *N. caninum* was constructed using the ITS1 rDNA gene sequence of our amplicon and those available in GenBank.

The *N. caninum* sequence described in this study (MW551566) was 99.5–100% homologous to *N. caninum* sequences published in the GenBank. The BLAST comparison of the partial sequences of the ITS1 rDNA gene showed that our sequence shares 100% homology with *N. caninum* from USA dog's (AY665715), Brazil cattle's (KY609325 and FJ966043), Southern Chile cattle's (KF536906), Iran dog's (KC710321), China dairy cows and pig's (JN634857, MF802344 and MK405580) and Australia dog's (MK203863 and KU253801). Our sequence shares 99.5% homology with *N. caninum* from seabirds from Brazil (MW044668 and MW023246), and pigs from China (MK751596).

### 3.3 Histopathology

Neither microscopic lesions nor *N. caninum* cysts were observed in all tissue‐stained samples examined under microscope.

## 4 DISCUSSION

*Neospora caninum* has been documented to infect most domestic wildlife but is known from primarily infect dogs and cattle and is considered an important cause of abortion in camels (Hosseininejad et al., 2009; Wolf et al., 2005).

Little is known about the presence of *N. caninum* in meat tissues from naturally infected camels in North Africa. To our knowledge, this is the first molecular report of *N. caninum* in Tunisian slaughtered camels.

In Southern Tunisia, camels are an important source of meat, leather and labour. They are also important source of wealth and social status. Studies detecting *N. caninum* DNA in Tunisian have been conducted in naturally infected goats, sheep and cattle (Amdouni et al., 2018a, 2018b, 2019) but there are no data about Tunisian camels.

Anti‐*N. caninum* antibodies were detected in various ruminants’ species worldwide. Seroprevalence reaches 87% in cattle, it ranges from 0 to 64% in sheep, 0 to 26.6% in goats and 3.7 to 86% in camels (Dubey & Schares, 2007; Selim & Abdelhady, 2020). For camels, seroprevalence was varied between 11.1 and 86% (Bártová et al., 2017; Hamidinejat et al., 2013; Hilali et al., 1998; Mentaberre et al., 2013; Mohammed et al., 2020; Nazir et al., 2017; Sadrebazzaz et al., 2006; Selim and Abdelhady, 2020). The highest prevalence of anti‐*N. caninum* antibodies was reported in Canary Islands (86%) by Mentaberre et al. (2013) explained by the absence of the definitive host in the surveyed farms, resulting in the vertical transmission of this parasite in camels, the same trend was reported by López‐Gatiu et al. (2004).

The molecular detection of *N. caninum* in camels reported in the study herein was greater than in Egypt at 24% (12/50) (Ahmed et al., 2017). Such differences of molecular detection can be attributed to differences in farming practices, hygienic measures, geographic factors and environmental influences (Dubey et al., 2007; Selim & Abdelhady, 2020).

Some findings have shown that *N. caninum* infection was positively correlated with age of animals and prevalence increase significantly in adult animals (Iovu et al., 2012; Nazir et al., 2017; Selim & Abdelhady, 2020). We found the same trend herein, the highest *N. caninum* molecular detection was found in animals of more than 3 years old (6/9) and in animals aged between more than 1 and 3 years old (4/12). Whilst, the lowest molecular detection (2/14) was observed in animals aged of 1 year or less (*p* = 0.035). This association with age can be explained by the repetitive cumulative infections and indicates the role of horizontal transmission in infection transmission (Selim & Abdelhady, 2020).

Several studies showed that *N. caninum* infection prevalence was statistically associated to gender (Bártová et al., 2017; Mentaberre et al., 2013; Selim & Abdelhady, 2020). No statistically significant difference in molecular detection according to gender of animals was found herein, this could be explained by the small sample size.

Infection caused by *N. caninum* was confirmed using nested PCR followed by sequencing of all amplicons. The sequence described in the current study (MW551566) shared high homology (99.5–100%) with sequences published in the GenBank. The alignment of the sequences with sequences published in the GenBank and the phylogenetic analysis showed 100% similarity with sequences from the United States, Brazil, Southern Chile, Iran, China and Australia. Sequence similarity may occur due to convergent evolution or by chance as the case with shorter sequences

## 5 CONCLUSION

The present findings provided an estimation of *N. caninum* molecular detection in Tunisian camels. To the best of our knowledge, this is the first report and molecular identification of *N. caninum* infection in Tunisian naturally infected camels. These result highlights the presence of a risk infection by *N. caninum* in camels. For preventing *N. caninum* infection further studies are needed to improve our knowledge about the epidemiology of neosporosis in North Africa.

## CONFLICT OF INTEREST

The authors declare no conflict of interest.

## AUTHOR CONTRIBUTIONS

Yosra Amdouni, Safa Amairia, Mohamed Gharbi, Amara Abdelkader and Imen abedennebi conceived and designed the experiments. Yosra Amdouni performed the experiments. Imen abedennebi and Walid Chandoul involved in the collection of samples. Yosra Amdouni and Mohamed Gharbi wrote and reviewed the manuscript.

## ETHICS STATEMENT

No ethical approval was required, as this study does not involve clinical trials. The camels involved in this study were slaughtered for human consumption.

## Supporting information




**Figure S1**: Phylogenetic tree of ITS1 rDNA gene for *Neospora caninum* isolated from tissues of Tunisian camelids and the other isolates deposited in GenBank. Sequence amplified in the present study is indicated with a black square.

Supporting information

## Data Availability

Data availability are available from the corresponding author

## References

[vms3901-bib-0001] Ahmed, N. , Al‐Akabway, L. , Ramadan, M. , Abd El‐Gawad, S. , & Moustafa, M. (2017). Serological and PCR‐sequencing assays for diagnosis of *Toxoplasma gondii* and *Neospora caninum* infecting camels in Egypt. Benha Veterinary Medical Journal, 33, 200–210. 10.21608/bvmj.2017.30466

[vms3901-bib-0002] Almería, S. , & López‐Gatius, F. (2013). Bovine neosporosis: Clinical and practical aspects. Research in Veterinary Science, 95(2), 303–309. 10.1016/j.rvsc.2013.04.008 23659742

[vms3901-bib-0003] Amdouni, Y. , Rjeibi, M. R. , Awadi, S. , Rekik, M. , & Gharbi, M. (2018a). First detection and molecular identification of *Neospora caninum* from naturally infected cattle and sheep in North Africa. Transboundry and Emerging Diseases, 65, 976–982. 10.1111/tbed.12828 29417744

[vms3901-bib-0004] Amdouni, Y. , Amairia, S. , Said, Y. , Awadi, S. , & Gharbi, M. (2018b). First molecular detection and phylogenetic analysis of *Neospora caninum* DNA from naturally infected goats in Northwest Tunisia. Acta Parasitologica, 63, 709–714. 10.1515/ap-2018-0083 30367762

[vms3901-bib-0005] Amdouni, Y. , Rouatbi, M. , Lassoued, N. , Rekik, M. , & Gharbi, M. (2019). *Neospora caninum* natural infection in Tunisian rams: Serological study and molecular identification of infection in semen. Acta Parasitological, 64(4), 821–828. 10.2478/s11686-019-00105-0 31418166

[vms3901-bib-0006] Ancelle, T. (2006). Statistique épidémiologie (2nd ed., Vol. 300, pp. 197–199). MALOINE.

[vms3901-bib-0007] Bártová, E. , Kobédová, K. , Lamka, J. , Kotrba, R. , Vodička, R. , & Sedlák, K. (2017). Seroprevalence of *Neospora caninum* and *Toxoplasma gondii* in exotic ruminants and camelids in the Czech Republic. Parasitology Research, 116, 1925–1929. 10.1007/s00436-017-5470-6 28497227

[vms3901-bib-0008] Buxton, D. , Maley, S. W. , Wright, S. , Thomson, K. M. , Rae, A. G. , & Innes, E. A. (1998). The pathogenesis of experimental neosporosis in pregnant sheep. Journal of Comparative Pathology, 118, 267–279. 10.1016/S0021-9975(07)80003-X 9651804

[vms3901-bib-0009] Chávez‐Velásquez, A. , Álvarez‐García, G. , Collantes‐Fernández, E. , Casas‐Astos, E. , Rosadio‐Alcántara, R. , Serrano‐Martínez, E. , & Ortega‐Mora, L. M. (2004). First report of *Neospora caninum* infection in adult alpacas (Vicugna pacos) and llamas (Lama glama). Journal of Parasitology, 90, 864–866. 10.1645/GE-260R 15357084

[vms3901-bib-0010] Donahoe, S. L. , Lindsay, S. A. , Krockenberger, M. , Phalen, D. , & Šlapeta, J. (2015). A review of neosporosis and pathologic findings of *Neospora caninum* infection in wildlife. International Journal for Parasitology: Parasites and Wildlife, 4, 216–238. 10.1016/j.ijppaw.2015.04.002 25973393 PMC4427759

[vms3901-bib-0011] Dubey, J. (1999). Neosporosis—the first decade of research. International Journal of Parasitology, 29, 1485–1488. 10.1016/S0020-7519(99)00134-4 10608433

[vms3901-bib-0012] Dubey, J. P. (2003). Review of *Neospora caninum* and neosporosis in animals. Korean Journal of Parasitology, 41, 1–16. 10.3347/kjp.2003.41.1.1 12666725 PMC2717477

[vms3901-bib-0013] Dubey, J. P. , & Schares, G. (2011). Neosporosis in animals‐The last five years. Veterinary Parasitology, 180, 90–108. 10.1016/j.vetpar.2011.05.031 21704458

[vms3901-bib-0014] Dubey, J. P. , Hemphill, A. , Calero‐Bernal, R. , & Schares, G. (2017). Neosporosis in animals (Vol. 530, pp. 133–336). CRC Press.

[vms3901-bib-0015] Dubey, J. P. , & Schares, G. (2007). Epidemiology and control of neosporosis and *Neospora caninum* . Clinical Microbiology Reviews, 20, 323–367. 10.1128/CMR.00031-06 17428888 PMC1865591

[vms3901-bib-0016] Dubey, J. P. , Schares, G. , & Ortega‐Mora, L. M. (2007). Epidemiology and control of neosporosis and N*eospora caninum* . Clinical Microbiology Reviews, 20, 323–367. 10.1128/CMR.00031-06 17428888 PMC1865591

[vms3901-bib-0017] Hamidinejat, H. , Ghorbanpour, M. , Rasooli, A. , Nouri, M. , Hekmatimoghaddam, S. , Namavari, M. M. , Pourmehdi‐Borojeni, M. , & Sazmand, A. (2013). Occurrence of anti‐*Toxoplasma gondii* and *Neospora caninum* antibodies in camels (Camelus dromedarius) in the center of Iran. Turkish Journal of Veterinary and Animal Sciences, 37, 277–281. 10.3906/vet-1110-21

[vms3901-bib-0018] Hässig, M. , Sager, H. , Reitt, K. , Ziegler, D. , Strabel, D. , & Gottstein, B. (2003). *Neospora caninum* in sheep: A herd case report. Veterinary Parasitology, 117, 213–220. 10.1016/j.vetpar.2003.07.029 14630429

[vms3901-bib-0019] Hilali, M. , Romand, S. , Thulliez, P. , Kwok, O. C. H. , & Dubey, J. P. (1998). Prevalence of *Neospora caninum* and *Toxoplasma gondii* antibodies in sera from camels from Egypt. Veterinary Parasitology, 75, 269–271. 10.1016/S0304-4017(97)00181-7 9637230

[vms3901-bib-0020] Hosseininejad, M. , Pirali‐Kheirabadi, K. , & Hosseini, F. (2009). Seroprevalence of *Neospora caninum* infection in camels (Camelus dromedarius) in Isfahan Province, center of Iran. Iranian Journal of Parasitology, 5, 1–21.

[vms3901-bib-0021] Ibrahim, A. M. , Ismail, A. A. , Elkhansa, T. , & Angara, E. (2014). Seroprevalence of *Neospora caninum* in dairy cattle and the co‐herded camels, sheep and goats in dairy farms in the Khartoum State, Sudan. Journal of Applied and Industrial Sciences, 2, 206–212.

[vms3901-bib-0022] Iovu, A. , Györke, A. , Mircean, V. , Gavrea, R. , & Cozma, V. (2012). Seroprevalence of *Toxoplasma gondii* and *Neospora caninum* in dairy goats from Romania. Veterinary Parasitology, 186, 470–474. 10.1016/j.vetpar.2011.11.062 22177331

[vms3901-bib-0023] López‐Gatiu, F. , Pabón, M. , & Almería, S. (2004). *Neospora caninum* infection does not affect early pregnancy in dairy cattle. Theriogenology, 62, 606–613. 10.1016/j.theriogenology.2003.11.002 15226015

[vms3901-bib-0024] McAllister, M. M. , McGuire, A. M. , Jolley, W. R. , Lindsay, D. S. , Trees, A. J. , & Stobart, R. H. (1996). Experimental neosporosis in pregnant ewes and their offspring. Veterinary Pathology, 33, 647–655. 10.1177/030098589603300603 8952023

[vms3901-bib-0025] Mentaberre, G. , Gutiérrez, C. , Rodríguez, N. F. , Joseph, S. , González‐Barrio, D. , Cabezón, O. , de la Fuente, J. , Gortazar, C. , & Boadella, M. (2013). A transversal study on antibodies against selected pathogens in dromedary camels in the Canary Islands, Spain. Veterinary Microbiology, 167, 468–473. 10.1016/j.vetmic.2013.07.029 23992795

[vms3901-bib-0026] Mohammed, O. B. , Amor, N. , Omer, S. A. , & Alagaili, A. N. (2020). Seroprevalence of toxoplasma gondii and neospora caninum in dromedary camels (Camelus dromedarius) from Saudi Arabia. Revista Brasileira de Parasitologia Veterinária, 29, 1–8. 10.1590/s1984-29612020008 32236334

[vms3901-bib-0027] Moreno, B. , Collantes‐Fernández, E. , Villa, A. , Navarro, A. , Regidor‐Cerrillo, J. , & Ortega‐Mora, L. M. (2012). Occurrence of *Neospora caninum* and *Toxoplasma gondii* infections in ovine and caprine abortions. Veterinary Parasitology, 187, 312–318. 10.1016/j.vetpar.2011.12.034 22260901

[vms3901-bib-0028] Nazir, M. M. , Oneeb, M. , Ayaz, M. M. , Bibi, F. , Ahmad, A. N. , Waheed, A. , Sajid, M. A. , Sultan, M. T. , Yasin, G. , & Lindsay, D. S. (2017). Prevalence of antibodies to *Neospora caninum* in the serum of camels (Camelus dromedarius) from central Punjab, Pakistan. Tropical Animal Health Production, 49, 1081–1084. 10.1007/s11250-017-1300-1 28470581

[vms3901-bib-0029] OEP . (2021). L'Office de l'Elevage et des Paturages ‐ Données sectorielles‐ 2017 ‐ Effectifs du cheptel [WWW Document]. http://www.oep.nat.tn/index.php/fr/donnees‐sectorielles‐/40‐effectif‐s‐du‐cheptel

[vms3901-bib-0030] Peters, M. , Wohlsein, P. , Knieriem, A. , & Schares, G. (2001). *Neospora caninum* infection associated with stillbirths in captive antelopes (Tragelaphus imberbis). Veterinay Parasitology, 97, 153–157. 10.1016/S0304-4017(01)00401-0 11358631

[vms3901-bib-0031] Reichel, M. P. , Alejandra Ayanegui‐Alcérreca, M. , Gondim, L. F. P. , & Ellis, J. T. (2013). What is the global economic impact of *Neospora caninum* in cattle ‐ The billion dollar question. International Journal for Parasitology, 43, 133–142. 10.1016/j.ijpara.2012.10.022 23246675

[vms3901-bib-0032] Sadrebazzaz, A. , Haddadzadeh, H. , & Shayan, P. (2006). Seroprevalence of Neospora caninum and Toxoplasma gondii in camels (Camelus dromedarius) in Mashhad, Iranian Journal of Parasitology, 98, 600–601. 10.1007/s00436-005-0118-3 16425066

[vms3901-bib-0033] Saitou, N. , & Nei, M. (1987). The neighbour‐joining method: A new method for reconstructing phylogenetic trees. Molecular Biology and Evolution, 4, 406–425. 10.1093/oxfordjournals.molbev.a040454 3447015

[vms3901-bib-0034] Schwartz, D. (1993). Méthodes statistiques à l'usage des médecins et des biologistes (3ème éd., Vol. 83, pp. 170–181). Flammarion.

[vms3901-bib-0035] Selim, A. , & Abdelhady, A. (2020). Neosporosis among Egyptian camels and its associated risk factors. Tropical Animal Health and Production, 52, 3381–3385. 10.1007/s11250-020-02370-y 32929587

[vms3901-bib-0036] Tamura, K. , Peterson, D. , Peterson, N. , Stecher, G. , Nei, M. , & Kumar, S. (2011). MEGA5: Molecular evolutionary genetics analysis using maximum likelihood, evolutionary distance, and maximum parsimony methods. Molecular Biology and Evolution, 28, 2731–2739. 10.1093/molbev/msr121 21546353 PMC3203626

[vms3901-bib-0037] Wang, Y. , Tian, R. M. , Gao, Z. M. , Bougouffa, S. , & Qian, P. Y. (2014). Optimal eukaryotic 18S and universal 16S/18S ribosomal RNA primers and their application in a study of symbiosis. PLoS One, 9, e90053. 10.1371/journal.pone.0090053 24594623 PMC3940700

[vms3901-bib-0038] Wolf, D. , Schares, G. , Cardenas, O. , Huanca, W. , Cordero, A. , Bärwald, A. , Conraths, F. J. , Gauly, M. , Zahner, H. , & Bauer, C. (2005). Detection of specific antibodies to *Neospora caninum* and *Toxoplasma gondii* in naturally infected alpacas (Lama pacos), llamas (Lama glama) and vicuñas (Lama vicugna) from Peru and Germany. Veterinary Parasitology, 130, 81–87. 10.1016/j.vetpar.2005.03.024 15893073

