# Supplementary figures and images for: First molecular detection of Neospora caninum from naturally infected slaughtered camels in Tunisia
Source: Vet Med Sci. 2022 Aug 16;8(5):2241–7. doi: 10.1002/vms3.901 (PMC9514472; doi:10.1002/vms3.901)

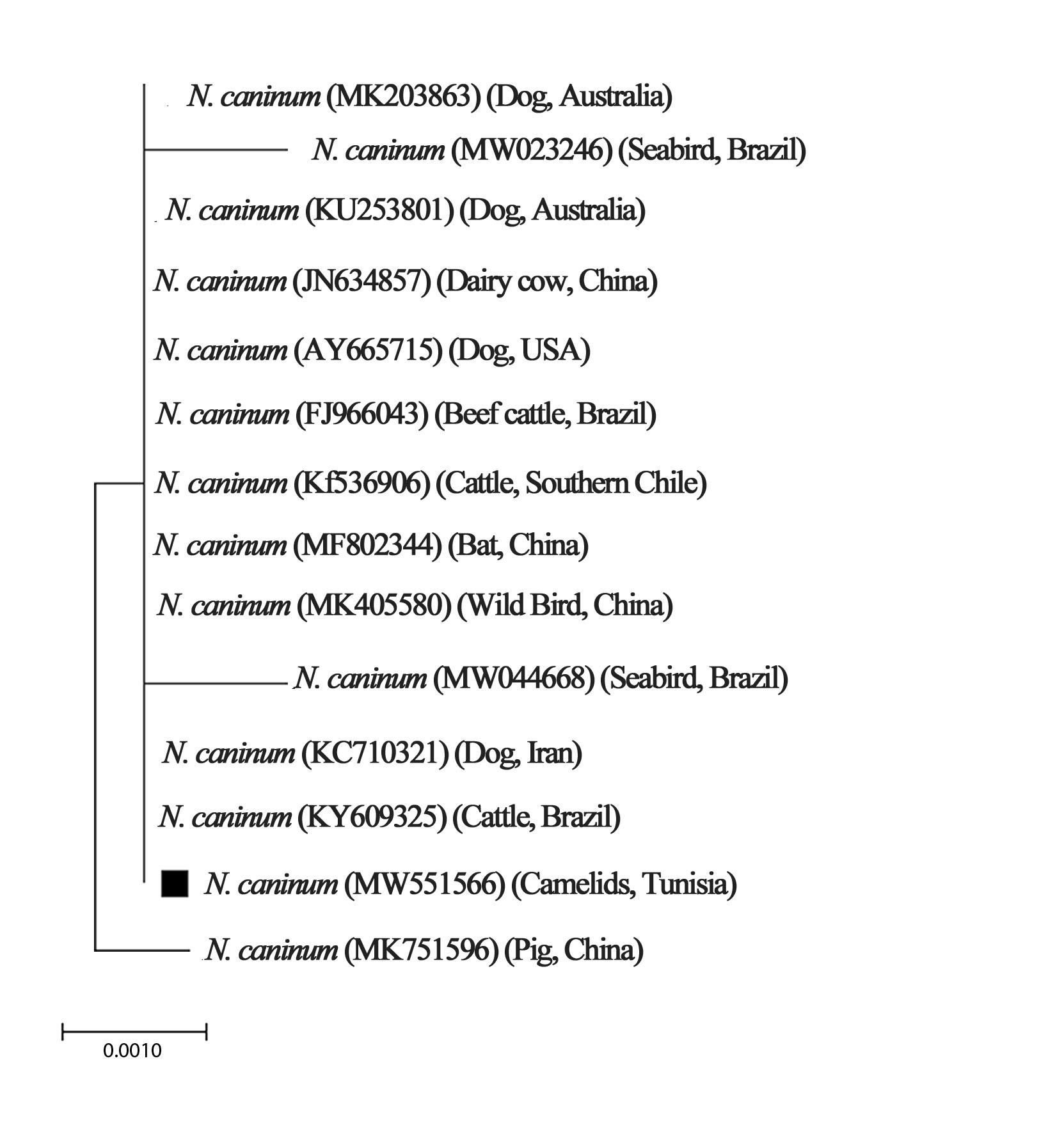

Supplement: Supplementary file 1 — Figure S1: Phylogenetic tree of ITS1 rDNA gene for Neospora caninum isolated from tissues of Tunisian camelids and the other isolates deposited in GenBank. Sequence amplified in the present study is indicated with a black square. [file VMS3-8-2241-s001.jpg]
