# Supplementary material for: First molecular detection of Neospora caninum from naturally infected slaughtered camels in Tunisia
Source: Vet Med Sci. 2022 Aug 16;8(5):2241–7. doi: 10.1002/vms3.901 (PMC9514472; doi:10.1002/vms3.901)
Supplement: Supplementary file 2 — Supporting information [file VMS3-8-2241-s002.docx]

**Table S1 : Universal PCR and *Neospora caninum* primers with their respective PCR conditions**

| **Target DNA** | **Target gene** | **Primer name, sequence (5′–3′)** | **Amplicon size (bp)** | | **Amplification cycle** | **Reference** |
| --- | --- | --- | --- | --- | --- | --- |
| Universal DNA | 18S | 1A: AACCTGGTTGATCCTGCCAGT | 700 | Initial denaturation: 5 min at 94°C  Amplification (25 cycles): 94°C; 59°C and 72°C for 50 s each  Final extension: 72°C for 10 min | | (Wang et al., 2014) |
|  |  | 564R: GGCACCAGACTTGCCCTC |  |  |  |  |
| *Neospora caninum* DNA | ITS1 | NN1: TCAACCTTTGAATCCCAA | 279 | **1^st^ PCR**  Initial denaturation: 5 min at 95°C  Amplification (26 cycles): 94°C, 48°C and 72°C for 1 min each  Final extension: 72°C for 5 min  **2^nd^ PCR**  Initial denaturation: 5 min at 95°C  Amplification (26 cycles): 94°C for 60 sec, 48°C for 30 sec and 30 sec at 72°C  Final extension: 72°C for 5 min | | (Buxton et al., 1998) |
|  |  | NN2: CGAGCCAAGACATCCATT |  |  |  |  |
|  |  | NP1: TACTACTCCCTGTGAGTTG |  |  |  |  |
|  |  | NP2: TCTCTTCCCTCAAACGCT |  |  |  |  |
